# IL-25 and IL-33 induce Type 2 inflammation in basophils from subjects with allergic asthma

**DOI:** 10.1186/s12931-016-0321-z

**Published:** 2016-01-14

**Authors:** Brittany M. Salter, John Paul Oliveria, Graeme Nusca, Steve G. Smith, Damian Tworek, Patrick D. Mitchell, Rick M. Watson, Roma Sehmi, Gail M. Gauvreau

**Affiliations:** Department of Medicine, McMaster University, HSC 3U26, 1200 Main St West, Hamilton, ON Canada; Department of Internal Diseases, Asthma and Allergy, Medical University of Lodz, Lodz, Poland

**Keywords:** Allergic Asthma, Basophils, Alarmin Cytokines, IL-25, IL-33

## Abstract

**Background:**

The alarmin cytokines IL-25 and IL-33 are key promoters of type 2 inflammation. Basophils respond to alarmin cytokines, however the relationship of these cytokines with basophil activation and recruitment in human studies of allergic asthma has not been well characterized. This study investigated the effect of IL-25 and IL-33 on basophils in a model of allergic asthma.

**Methods:**

10 mild allergic asthmatics underwent allergen and diluent inhalation challenges. Bone marrow aspirates were collected at pre-challenge and 24 h (h) post challenge. Peripheral blood and sputum samples were collected at pre-challenge, 7 h, and 24 h post-challenge to measure basophil expression of IL-17RB, ST2, and intracellular IL-25. Freshly isolated peripheral blood basophils from allergic donors were incubated overnight with IL-25 and IL-33, or sputum supernatant collected post-allergen to assess pro-inflammatory effects of mediators released in the airways.

**Results:**

There were increased percentage of basophils expressing IL-17RB, ST2, and intracellular IL-25 collected from bone marrow, peripheral blood, and sputum after allergen inhalation challenge. In vitro stimulation with IL-25 and IL-33 increased the percentage of basophils expressing intracellular type 2 cytokines and surface activation markers, and primed eotaxin-induced migratory potential of basophils, which was mediated directly through IL-17RB and ST2, respectively. Stimulation of basophils with sputum supernatants collected post-allergen challenge up-regulated the percentage of basophils expressing markers of activation and intracellular type 2 cytokines, which was reversed following blockade of the common β chain (βc).

**Conclusions:**

Our findings indicate that the alarmin cytokines IL-33 and IL-25 increase basophil activation and migratory potential, and may pose as a novel therapeutic targets for the treatment of allergic asthma.

**Electronic supplementary material:**

The online version of this article (doi:10.1186/s12931-016-0321-z) contains supplementary material, which is available to authorized users.

## Background

Asthma is a chronic respiratory disease characterized by reversible airway obstruction, airway inflammation, and airway hyperresponsiveness (AHR). There are numerous causes of this disorder, and recent evidence indicates that the alarmin cytokines IL-25 and IL-33 may play a key role in promoting allergic asthma.

In mouse models, transgenic over-expression or administration of IL-25 and IL-33 generates airway eosinophilia, up-regulated Type 2 cytokine expression, elevated serum IgE, AHR and mucus hypersecretion [1–6]. Conversely, neutralization of IL-25 and IL-33 leads to reduction of airway inflammation, IgE levels, Type 2 cytokine expression, goblet cell hyperplasia, and AHR [7–10].

IL-25 and IL-33 have been shown to exert their effects on progenitor cells, mast cells, granulocytes, lymphocytes and dendritic cells [11–20]. In human studies of allergic asthma, IL-33 and ST2 expression in serum, lung tissue and BALF have found to be higher in asthmatics compared to healthy controls and correlate with asthma severity [21–25]. Similarly, levels of IL-25 and IL-17RB mRNA expression are elevated in serum, bronchial mucosa and the skin, which correlates with allergic disease severity [21–26].

Basophils are involved in the early asthmatic response (EAR) of asthma, acting as potent sources of histamine and cysteinyl leukotrienes, which contribute to bronchoconstriction. Emerging evidence suggests that basophils have an important role in promoting delayed airway inflammation. Peripheral and airway basophils increase in number and activation in the LAR following allergen inhalation, and are a significant source of IL-4 and IL-13 [27–30]. We have previously reported that the percentage of basophils expressing surface activation markers, intracellular cytokines (IL-4; IL-13) and TSLPR, significantly increase in peripheral blood and sputum post-allergen inhalation in mild allergic asthmatics for up to 24 h [28]. We have also shown in vitro that TSLP can promote human basophil pro-inflammatory activity and prime migratory potential to eotaxin [28]. Despite these findings, it is not well known what factors promote basophil lung-homing and effector activity during the LAR, but reports suggest that alarmin cytokines may play a role in regulating basophil activity. Protein and mRNA expression of ST2 and sST2 have been confirmed on basophils, and become up-regulated following stimulation with IL-33 or IL-3 [16, 31–34]. In vitro stimulation with IL-33 promotes basophil IgE-dependent and IgE-independent release of histamine, and secretion of IL-4, IL-8, IL-5, IL-9, IL-6, IL-13, MCP and MIP [16, 31–34]. IL-33 can induce human basophil CD11b expression, adhesion and prime eotaxin-induced migration, suggesting that IL-33 may regulate basophil lung-homing [31]. Human basophils produce IL-25 following IgE cross-linking and constitutively express IL-17RB, which can be up-regulated following IL-3 stimulation [35, 36]. Lastly, IL-25 can inhibit basophil apoptosis and enhance IgE-mediated degranulation [36].

Despite IL-33 and IL-25 being identified as important promoters of allergic inflammation in mouse models, the influence of these cytokines on the effector role of basophils in human allergic asthma needs to be further characterized. Furthermore, little is known about the basophil response to the airway microenvironment, including mediators released by the epithelium and other inflammatory cells, following allergen inhalation. The purpose of this study was to determine the relationship between basophils and alarmin cytokines in human allergic asthma, and to examine the basophil response to airway secretions following allergen inhalation in mild allergic asthmatics.

## Methods

### Allergen challenge study design

Ten subjects with mild allergic asthma underwent allergen and diluent inhalation challenges separated by a two-week wash out period (Table 1). All subjects had a positive skin prick test (wheals > 2 mm) to common aeroallergens, methacholine PC_20_ < 16 mg/mL, FEV_1_ of ≥70 % of predicted and a dual phase response to allergen (fall in FEV_1_ of ≥20 % by 2 h and ≥15 % 3–7 h post allergen). Subjects used short-acting bronchodilators for control of asthma and were excluded from the study if they were pregnant or nursing, current smokers or ex-smokers with more than 10 pack-years, or developed lower respiratory infections/exacerbations or used inhaled or oral steroids 4 weeks prior to study. Anti-histamines, caffeine and non-steroidal anti-inflammatory agents were prohibited 48 h before any study visit.

**Table 1:** Subject Characteristics of Mild Allergic Asthmatics

Sex	Age (y)	% Predicted FEV_1_	Allergen Inhaled	Titration of Inhaled Allergen
M	61	96	Cat	1:32
F	24	80	Ragweed	1:16
M	52	95	HDM	1:64
F	40	104	Cat	1:128
M	25	86	Ragweed	1:8
F	24	93	HDM	1:8
M	26	93	HDM	1:32
M	20	94	Grass	1:4
F	34	70	Cat	1:512
M	26	96	Cat	1:8
No. M/F 6:4	Mean 33.2 ± 13.6	Mean 90.7 ± 9.6		

*F* female, *M* male, *HDM* house dust mite. Data presented as mean ± SEM

Baseline samples of peripheral blood, bone marrow aspirate and sputum samples were collected on day 1 and subjects were randomized to diluent or allergen challenge on day 2. At 7 h post-challenge peripheral blood and sputum was collected and at 24 h post-challenge bone marrow, peripheral blood and sputum samples were collected. AHR to allergen was measured by a shift in methacholine PC_20_ measured before and 24 h after challenge. Subjects returned after another two-week wash out period to donate 100 mL of peripheral blood for in vitro airway sample experiments.

Basophils for the in vitro experiments were purified from 100 mL of peripheral blood of 8 donors, confirmed to be allergic through positive skin prick testing to common aeroallergens. The study was approved by Hamilton Integrated REB and all subjects provided signed informed consent.

### Methacholine challenge

The methacholine inhalation challenge was carried out via tidal breathing from a Wright nebulizer, as previously described [37]. In brief, doubling concentrations of methacholine chloride (Methapharm, ON) were inhaled orally from a Hans Rudolph valve for 2 min. The FEV_1_ was measured following inhalation at 30 s and 90 s or until it stopped falling. The percent fall was calculated from the post-diluent FEV_1_ value. The test was terminated when a fall in FEV_1_ of at least 20 % of the lowest post-saline value occurred. The methacholine PC_20_ was calculated using linear interpolation.

### Allergen and diluent challenge

Allergen challenges were conducted by administering inhaled doubling concentrations of aeroallergen extract, such as house dust mite, cat dander and pollen, as previously described [38]^.^ The extract giving the largest skin wheal was selected for inhalation and the concentration of allergen required to achieve a 20 % decrease in FEV_1_ (the allergen PC_20_) was predicted using the methacholine PC_20_ and skin test reactivity. Diluent challenges were conducted using 3 inhalations of 0.9 % saline for 2 min each. The EAR was the largest percent fall in FEV_1_ between 0 and 2 h, and the LAR was the largest percent fall in FEV_1_ between 3 and 7 h post-challenge.

### Bone marrow, blood, and sputum sample processing

During allergen and diluent inhalation challenges, 10 mL of peripheral blood was collected into sodium heparin vaccutainers and 10 mL of bone marrow was aspirated from the iliac crest into heparin (1,000 U/mL). To semi-isolate basophils from bone marrow, samples were diluted in McCoys 5A, layered on Lymphoprep and centrifuged at 2200 rpm for 20 min (min) at room temperature (RT).

Sputum samples were induced using hypertonic saline and mucous plugs were selected from samples followed by dispersement with dithiothreitol, as previously described [39]. Sputum samples were centrifuged at 790 g 4 °C for 10 min, followed by collection of supernatant, additional centrifugation of the supernatant at 1500 g 4 °C for 10 min, and aliquoting of supernatant into eppendorfs that were frozen at −80 °C. Cytospins were prepared from sputum cells and stained with Diff-Quik for differential cell counts and toluidine blue for metachromatic cell counts.

Commercially available multiplex immunoassay kits were used to measure IL-33 and IL-25 (Bio-Rad laboratories, Inc, CA, US) in bone marrow, peripheral blood and sputum samples collected pre and post-challenges. Peripheral blood, bone marrow and sputum cells were immunostained with isotype or specific monoclonal antibodies (mAbs) and acquired via flow cytometry to identify basophils and measure the percent cell expression of IL-17RB, ST2, and intracellular IL-25 (all R&D Systems, MN, US) (Additional file 1: Figure S1, Additional file 2: Figure S2 and Additional file 3: Figure S3).

### Purification of basophils for cell cultures

100 mL of blood was diluted with McCoys 5A media, and layered on Lymphoprep. Erythrocytes were lysed and basophils were purified via negative selection with magnetic separation beads (Stem Cell Technologies, BC, Canada). Basophil cell suspensions were >98 % pure as determined by flow cytometry and >90 % viable by trypan blue (Additional file 4: Figure S4).

### 18 h Basophil Cultures with IL-25 and IL-33

Basophils purified from peripheral blood of 8 allergic subjects were suspended in RPMI-C, then incubated for 18 h at 37 °C with monensin (Biolegend, CA, US), and PBS, IL-3 (R&D Systems, MN, US), anti-IgE (Sigma, MO, US), or a pre-determined optimal dose (Additional file 5: Figure S5, Additional file 6: Figure S6) of IL-33 or IL-25 (all 10 ng/mL) (R&D Systems, MN, US) and with isotype controls or neutralizing antibodies to ST2 or IL-17RB (both 10 μg/mL) (both R&D Systems, MN, US). Basophils were immunostained with isotype or specific mAbs to identify basophils (Additional 4: Figure S4) and measure expression of CD203c (MACS Miltenyi, CA, US), CCR3 (Ebioscience, CA, US), ST2 (Ebioscience, CA, US), IL-17RB, TSLPR (Ebioscience, CA, US), as well as intracellular IL-4 (Ebioscience, CA, US) and IL-13 (Ebioscience, CA, US) via flow cytometry.

### Shape change assay with IL-33 and IL-25

To determine if IL-33 and IL-25 had a direct effect on basophil shape change, purified basophils from allergic donors (*n* = 8) suspended in RPMI-C were incubated with PBS, IL-33 or IL-25 (both R&D Systems, MN, US) (0.1, 1, 10, 100 ng/mL) at 37 °C for 0, 30, 45, 60 or 180 seconds (s). To determine if IL-33 or IL-25 primed the basophil shape change response to eotaxin, purified basophils were incubated for 18 h at 37 °C in RPMI-C with PBS, IL-33 or IL-25 (all 10 ng/mL). Following incubation, cells were stimulated with/without eotaxin (R&D Systems, MN, US) (5 ng/mL) for 0, 30, 45, 60 or 180 s. Basophils were fixed with ice-cold 1 % PFA for 10 min to stop the reaction and cell shape change was measured by the parameter FSC via flow cytometry.

### 18 h basophil cultures with airway samples

Purified basophils resuspended in RPMI-C collected from the mild allergic asthmatic subjects were incubated for 18 h at 37 °C with monensin and sputum supernatant previously collected from allergen inhalations at baseline, 7 and 24 h post-challenge. To measure the effect of receptor blockade, isolated basophils were treated with 10 μg/mL isotype controls or neutralizing antibodies targeting the βc, TSLPR, IL-17RB, and ST2 (all R&D Systems, MN, US). Incubation with PBS served as the negative control, and incubation with 10 ng/mL IL-3 was the positive control. Basophils were immunostained to identify the basophil population and to measure both CD203c and intracellular expression of IL-13 and IL-4 by flow cytometry.

### Flow cytometry staining and analysis

Cells for flow cytometry experiments were immunostained with isotype or specific mAbs to the extracellular CD45 (Ebioscience, CA, US), HLA-DR (Ebioscience, CA, US), CCR3, TSLPR, IL-3Rα (Ebioscience, CA, US) and CD203c for 30 min at 4 °C. To measure intracellular cytokine expression, cells were washed, fixed and permeabilized, then stained with isotype controls or antibodies to IL-4, IL-13 and IL-25. Cells were washed and acquired with a LSR II flow cytometer (BD Biosciences, CA, US). Analyses were performed using Flow-Jo software (Tree Star, CA, US). Basophils were defined as the CD45^+^/HLA-DR^−^/IL-3Rα^+^ population, as outlined in Additional files 1, 2, 3 and 4. The isotype control for the markers of interest was set to 2 %, which was compared to the specific markers to detect the percentage of cells expressing the marker.

### Statistical analysis

All data are presented as mean ± SEM except for flow cytometry data which are expressed as median percent positive cells. Statistical analysis for inhalation challenges was performed using 2-way ANOVA and post-hoc Bonferroni test. In vitro experiments were analyzed using 1-way ANOVA with a *post-hoc* Tukey test. Correlation analyses were conducted using a Spearman correlation test. Significance was accepted at *P* < 0.05.

## Results

### Allergen inhalation induces airway bronchoconstriction, hyperresponsiveness and inflammation mild allergic asthmatics

Inhalation of allergen induced a maximum fall in FEV_1_ of 20.1 ± 5.5 % within the first 2 h, and a maximum fall in FEV_1_ of 20.8 ± 5.2 % 3–7 h post-challenge, compared to no change in FEV_1_ following diluent inhalation. The methacholine PC_20_ decreased one doubling dose post-allergen challenge, compared to no change post-diluent.

Compared to diluent, the allergen inhalation challenge induced a significant increase in the sputum total cell count and number of granulocytes (Table 2). IL-25 could be detected in the bone marrow of 2 subjects, peripheral blood of 7 subjects, and sputum of 3 subjects. IL-33 could be detected in the bone marrow of 6 subjects, peripheral blood of 4 subjects and sputum of 3 subjects. We found no significant change in IL-25 or IL-33 levels in bone marrow, peripheral blood or sputum after allergen challenge (data not shown). The measurable levels of IL-25 or IL-33 showed no correlation with allergen-induced change in AHR to methacholine or maximum fall in FEV_1_.

**Table 2:** Allergen inhalation induces airway inflammation in mild allergic asthmatics. Sputum cell count was measured pre and post-challenge to determine effect of allergen compared to diluent. Data are presented as mean ± SEM

	Pre-Diluent Challenge	7 h-Post Diluent Challenge	24 h-Post Diluent Challenge	Pre-Allergen Challenge	7 h-Post Allergen Challenge	24 h-Post Allergen Challenge
Sputum Total Cell Count (×10^4^ cells/mL)	1.7 ± 1.2	1.5 ± 0.9	1.5 ± 0.8	1.8 ± 1.2	3.4 ± 2.9	3.1 ± 2.3^a^
Neutrophils (×10^4^ cells/mL)	1.0 ± 1.3	1.2 ± 1.3	1.3 ± 1.4	0.9 ± 0.9	2.4 ± 2.3^a^	1.7 ± 1.3
Eosinophils (×10^4^ cells/mL)	0.01 ± 0.0	0.02 ± 0.0	0.006 ± 0.0	0.02 ± 0.0	0.3 ± 0.4^a^	0.3 ± 0.4^a^
Metachromatic cells (×10^4^ cells/mL)	0 ± 0.0	0 ± 0.0	0 ± 0.0	0.01 ± 0.0	0.2 ± 0.3^a^	0.2 ± 0.2^a^

^a^Significantly different than diluent control; *P* < 0.05

### Allergen inhalation induces changes in basophil expression of IL-17RB and ST2

**Fig. 1:**
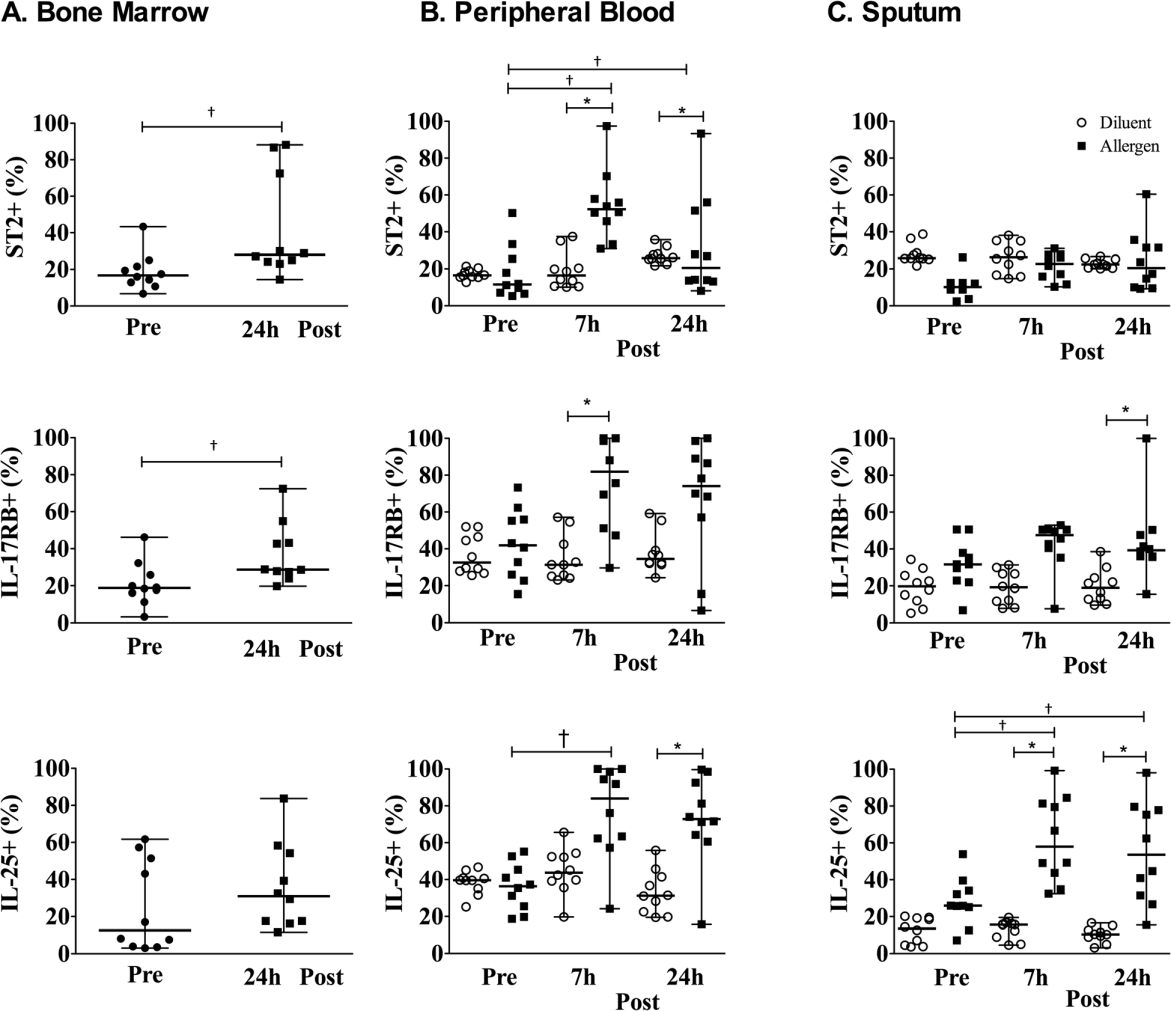
Allergen-inhalation induces changes in surface receptor expression for alarmin cytokines on basophils of mild allergic asthmatics. Percent of basophils expressing ST2, IL-17RB and intracellular IL-25 in 10 mild allergic asthmatics was measured in (**a**) bone marrow, (**b**) peripheral blood and (**c**) sputum at pre-allergen, 7 h and 24 h post-allergen (*black symbols*), compared to diluent (*open symbols*). Data are presented as median ± range. *Significantly different from pre-challenge for bone marrow and corresponding diluent-control time-point for peripheral blood and sputum, † significantly different from 0 h; *P* < 0.05 (*N* = 10)

**Fig. 2:**
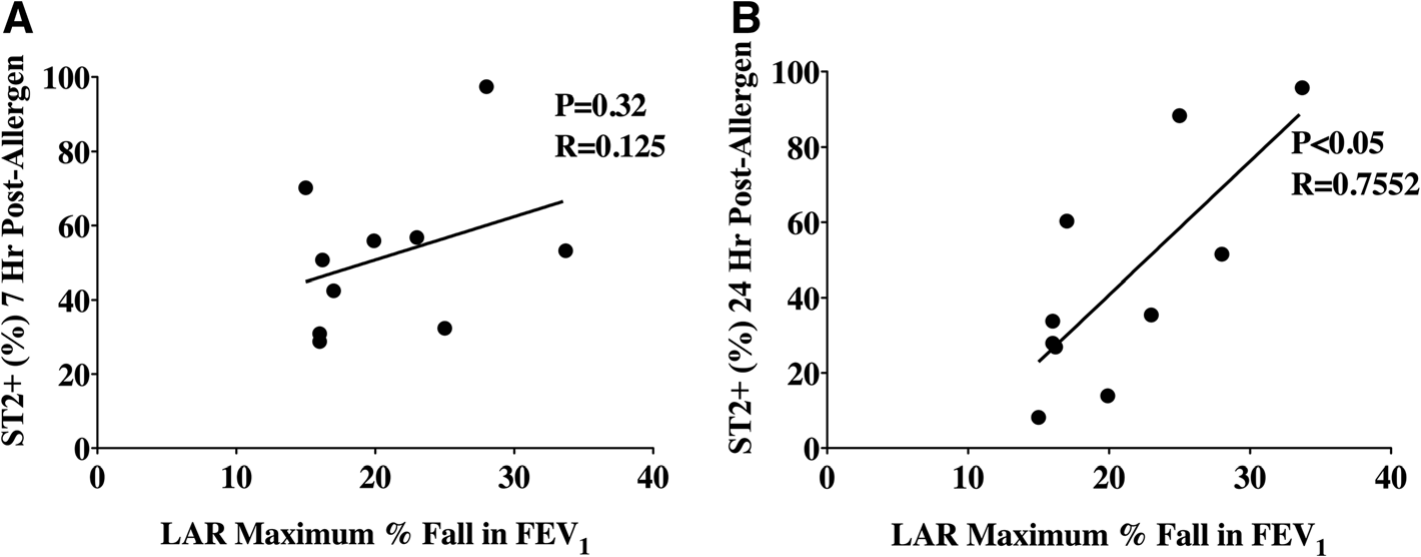
Correlation between ST2 basophil expression and lung function following allergen challenge in mild allergic asthmatics. Percent peripheral blood basophil expression in 10 mild allergic asthmatics of (**a**) ST2 at 7 h post-allergen compared to LAR, (**b**) ST2 at 24 h post-allergen compared to LAR. *Significant correlation between basophil ST2 expression and lung function; *P* < 0.05 (*N* = 10)

In bone marrow, there was a significant increase in the percentage of basophils expressing IL-17RB and ST2 at 24 h post-allergen challenge compared to baseline (Fig. 1a). In the peripheral blood we observed a significant increase in the percentage of basophils expressing IL-17RB at 7 h post-allergen and ST2 at 7 and 24 h post-allergen, compared to diluent (Fig. 1b). The percentage of peripheral blood basophils expressing ST2 at 7 and 24 h post-allergen was significantly greater compared to baseline (Fig. 1c). The percentage of sputum basophils expressing IL-17RB increased significantly at 24 h post-allergen, whereas no change was found with respect to ST2, compared to diluent (Fig. 1). Percentage of basophils expressing intracellular levels of IL-25 increased at 24 h post-allergen in peripheral blood and remained elevated for up to 24 h post-allergen in the sputum, compared to diluent (Fig. 1). Allergen did not change the percentage of basophils expressing intracellular IL-25 in the bone marrow. We observed a significant positive correlation between allergen-induced maximum % fall FEV_1_ 3–7 h and percentage of peripheral blood basophils expressing ST2 measured 24 h post-allergen (*P* = 0.015, *R* = 0.75) (Fig. 2b). No relationship was found between allergen-induced methacholine PC_20_ and peripheral blood ST2, IL-17RB or intracellular IL-25 basophil expression. Lastly, no correlation was found between airway ST2, IL-17RB or intracellular IL-25 basophil expression and lung function (methacholine PC_20_; LAR FEV_1_).

### Basophils express functional receptors for IL-33 and IL-25

**Fig. 3:**
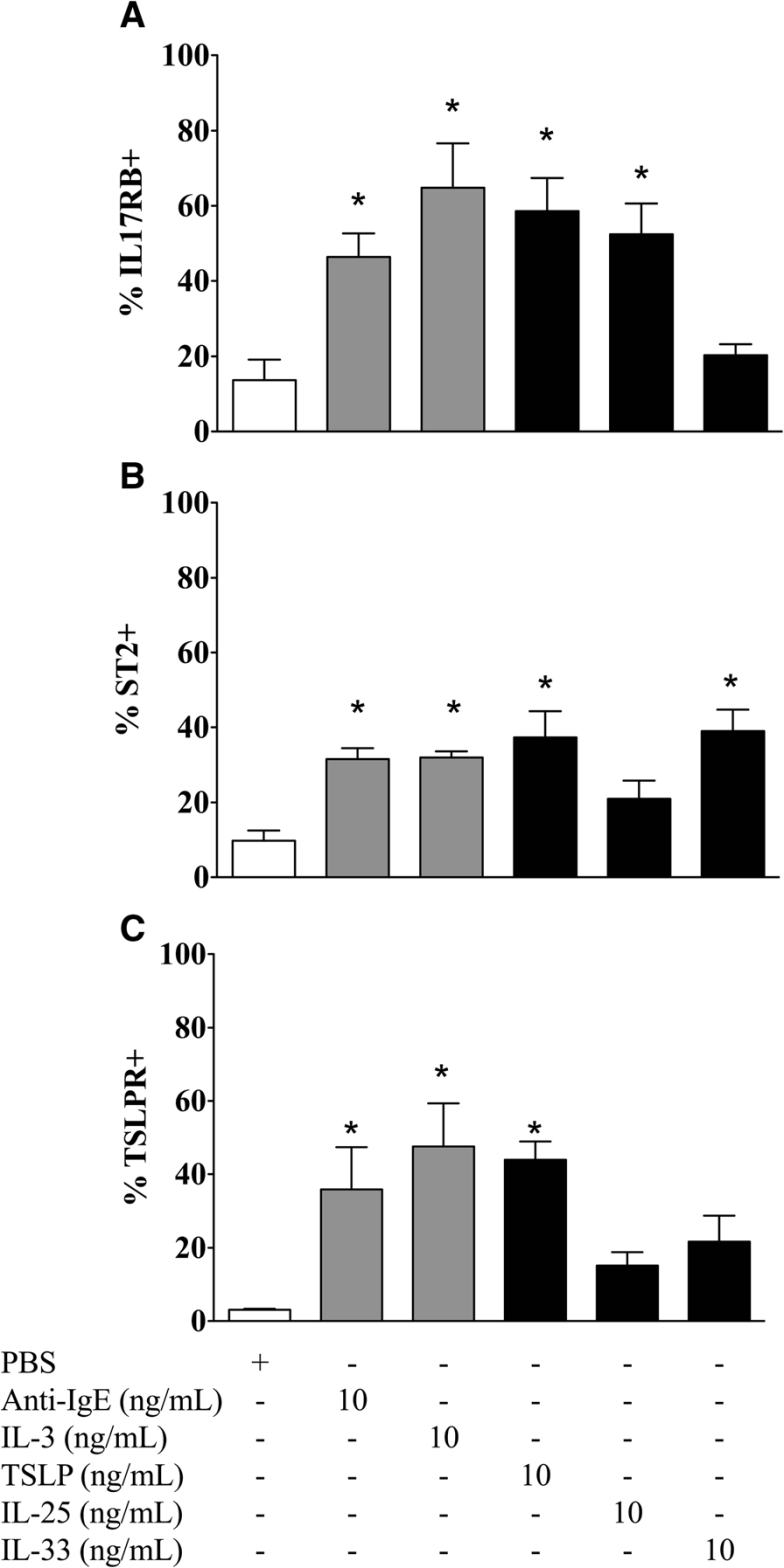
IL-25 and IL-33 up-regulate expression of their own receptor on basophils. Purified peripheral blood basophils from 8 allergic donors were suspended in RPMI-C and cultured for 18 h with PBS or optimal concentrations of anti-IgE, IL-3, TSLP, IL-25, or IL-33 (all 10 ng/mL), then assessed for percent basophil expression of (**a**) IL-17RB, (**b**) ST2, and (**c**) TSLPR. Data are expressed as mean ± SEM. *Significantly different from PBS negative control; *P* < 0.05 (*N* = 8)

Basophils from allergic subjects expressed IL-17RB and ST2, and stimulation with IL-3 and anti-IgE significantly increased the percentage of basophils expressing these receptors (Fig. 3a, b). Stimulation with IL-25 and IL-33 markedly up-regulated the percentage of basophils expressing receptors for these cytokines, IL-17RB and ST2, respectively (Fig. 3a, b). IL-25 had no effect on percent cell expression of ST2, nor did IL-33 affect IL-17RB (Fig. 3b). IL-25 and IL-33 had no effect on TSLPR expression, however TSLP increased the percentage of basophils expressing IL-17RB and ST2 (Fig. 3c).

### IL-33 and IL-25 exert pro-inflammatory effects on basophils

**Fig. 4:**
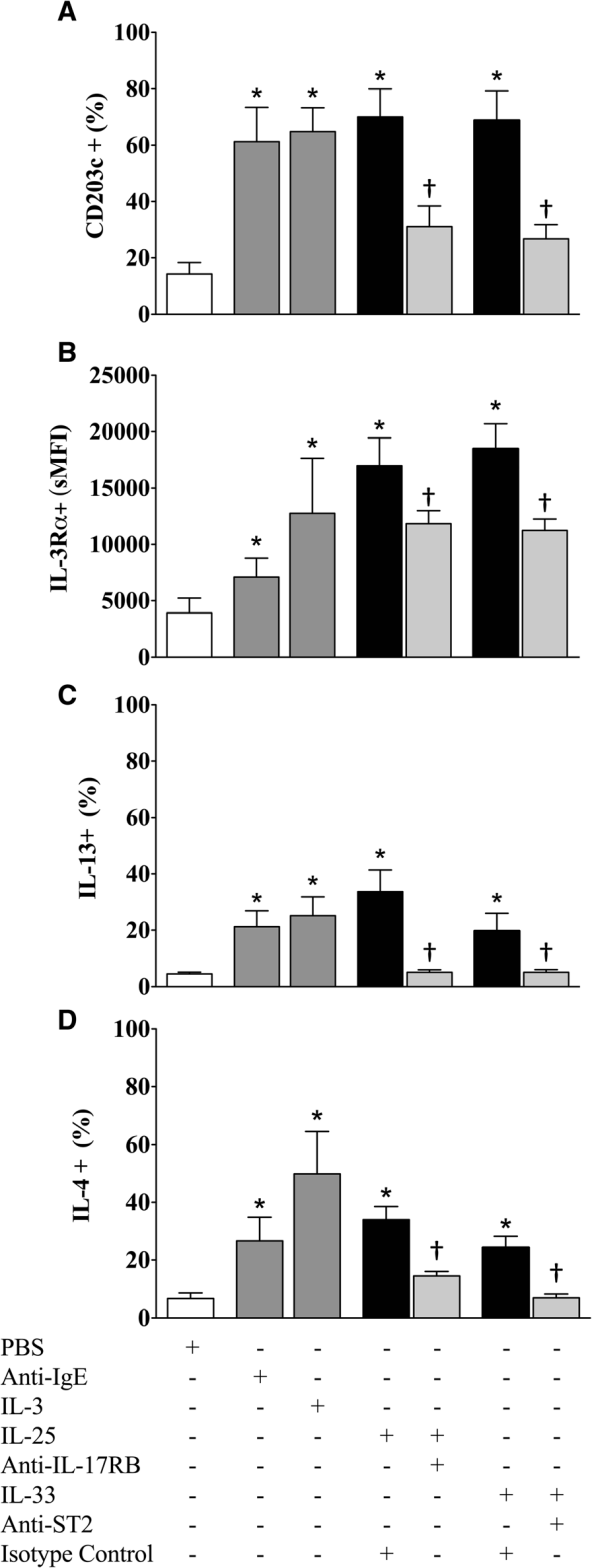
The effects of IL-25 and IL-33 on markers of basophil activation. Purified peripheral blood basophils from 8 allergic donors were incubated for 18 h with or without IL-25 or IL-33 (both 10 ng/mL) and neutralizing antibodies for IL-17RB and ST2 or isotype controls (both 10 μg/mL). Receptor blockade (light grey bars) was assessed by comparison to isotype controls (black bars) via measuring: (**a**) CD203c, (**b**) IL-3Rα, (**c**) intracellular IL-13, and (**d**) intracellular IL-4. Anti-IgE and IL-3 (10 ng/mL) served as positive controls (dark grey bars). Data are expressed as mean ± SEM. *Significantly different from PBS negative control, † significantly different from isotype control *P* < 0.05 (*N* = 8)

We examined the effect of 18 h incubation with IL-25 and IL-33 on markers of basophil activation and observed a significantly higher percentage of basophils expressing intracellular IL-4 and IL-13, as well as CD203c (Fig. 4). The level of IL-3Rα basophil expression increased, as shown by significant shift in the sMFI (Fig. 4). The aforementioned measurements significantly decreased following treatment with neutralizing antibodies to IL-17RB and ST2.

### IL-25 and IL-33 prime basophil migratory potential in response to eotaxin

**Fig. 5:**
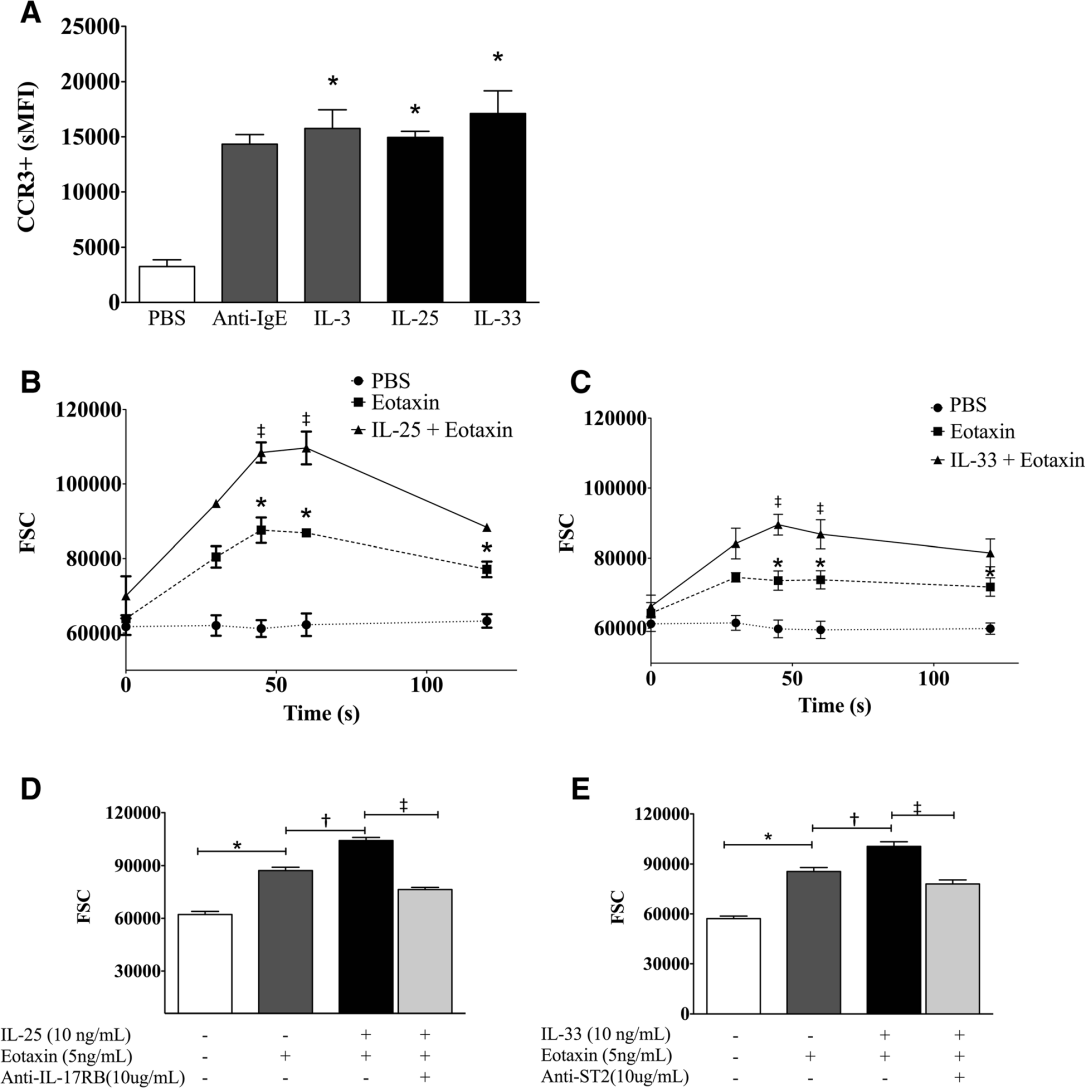
IL-25 and IL-33 prime eotaxin-induced basophil shape change. Purified peripheral blood basophils from 8 allergic donors were suspended in RPMI-C and incubated with/without IL-25 and IL-33 (10 ng/mL) to assess (**a**) CCR3 expression and (**b**, **c**) shape change was measured via change in FSC post-stimulation with eotaxin (5 ng/mL). The effect of neutralizing antibodies for (**d**) IL-17RB and (**e**) ST2 (light grey bars) on shape change was also assessed. Data are expressed as mean ± SEM. ‡ Significantly different from eotaxin, *significantly different from PBS negative control, † significantly different from isotype control; *P* < 0.05 (*N* = 8)

Compared to PBS negative control, incubation with IL-33 and IL-25 for 18 h induced significant up-regulation of CCR3 expression on basophils, as shown by a shift in the sMFI (Fig. 5a). IL-33 and IL-25 did not directly stimulate basophil shape change (data not shown), however overnight incubation with IL-25 and IL-33 induced greater shape change in response to stimulation with eotaxin (Fig. 5b, c). The priming effect was observed by 45 s following eotaxin stimulation and maintained for up to 180 s, and treatment with neutralizing antibodies to IL-17RB and ST2 significantly inhibited the priming effects of IL-33 and IL-25 on eotaxin-induced basophil shape change compared to the isotype control (Fig. 5d, e).

### Sputum supernatant samples collected post-allergen inhalation induce basophil activation

**Fig. 6:**
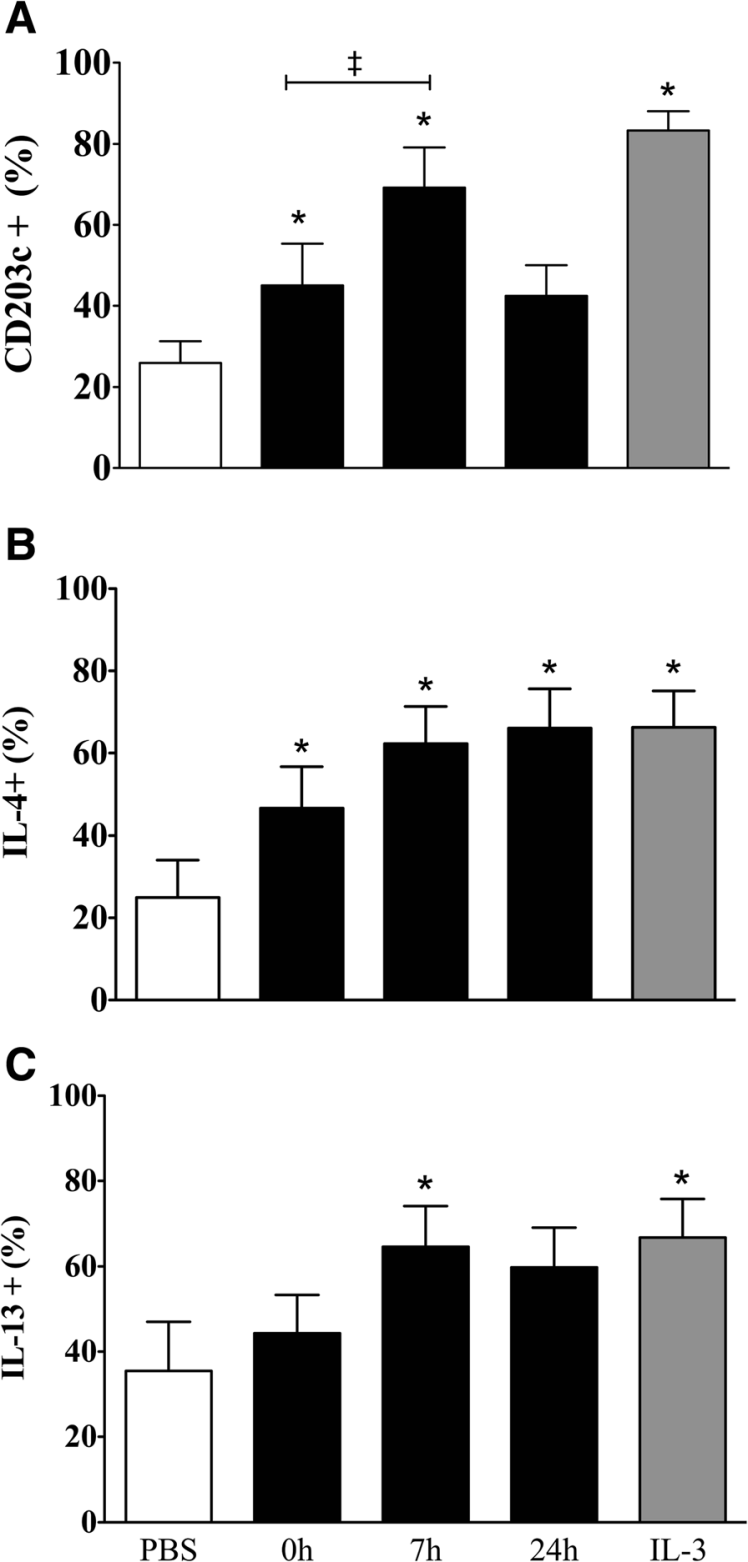
The effects of airway samples on markers of basophil activation. Purified peripheral blood basophils from 8 mild allergic asthmatic subjects were suspended in RPMI-C and cultured for 18 h with PBS as negative control (open bars) or IL-3 (10 ng/mL) as positive control (dark grey bars) or sputum supernatant collected from dual responders at 0 h, 7 h, and 24 h post-allergen (black bars) then measured for expression of (**a**) CD203c, (**b**) IL-4, and (**c**) IL-13. Data expressed as mean ± SEM. *Significantly different from PBS negative control; *P* < 0.05 (*N* = 8)

Commercial multiplex immunoassays were unable to detect a complete data set of IL-33 and IL-25 levels in airway samples. As a result, we used an alternative bioassay to evaluate the in vitro effect of airway secretions collected after allergen inhalation on basophil activation. Airway samples collected 7 h post-allergen had the greatest effect on basophil activity as shown by increased percentage of basophils expressing CD203c, and intracellular IL-4 and IL-13 compared to PBS negative control and 0 h samples (Fig. 6a). There was no difference between the effect of IL-3 (positive control) and 7 h post-allergen airway samples on the percentage of basophils expressing CD203c and intracellular IL-4 and IL-13. Incubation with 0 h airway samples significantly increased the percentage of basophils expressing CD203c and intracellular IL-4 (Fig. 6a, b), whereas 24 h airway samples increased basophil percent expression of intracellular IL-4 (Fig. 6b).

**Fig. 7:**
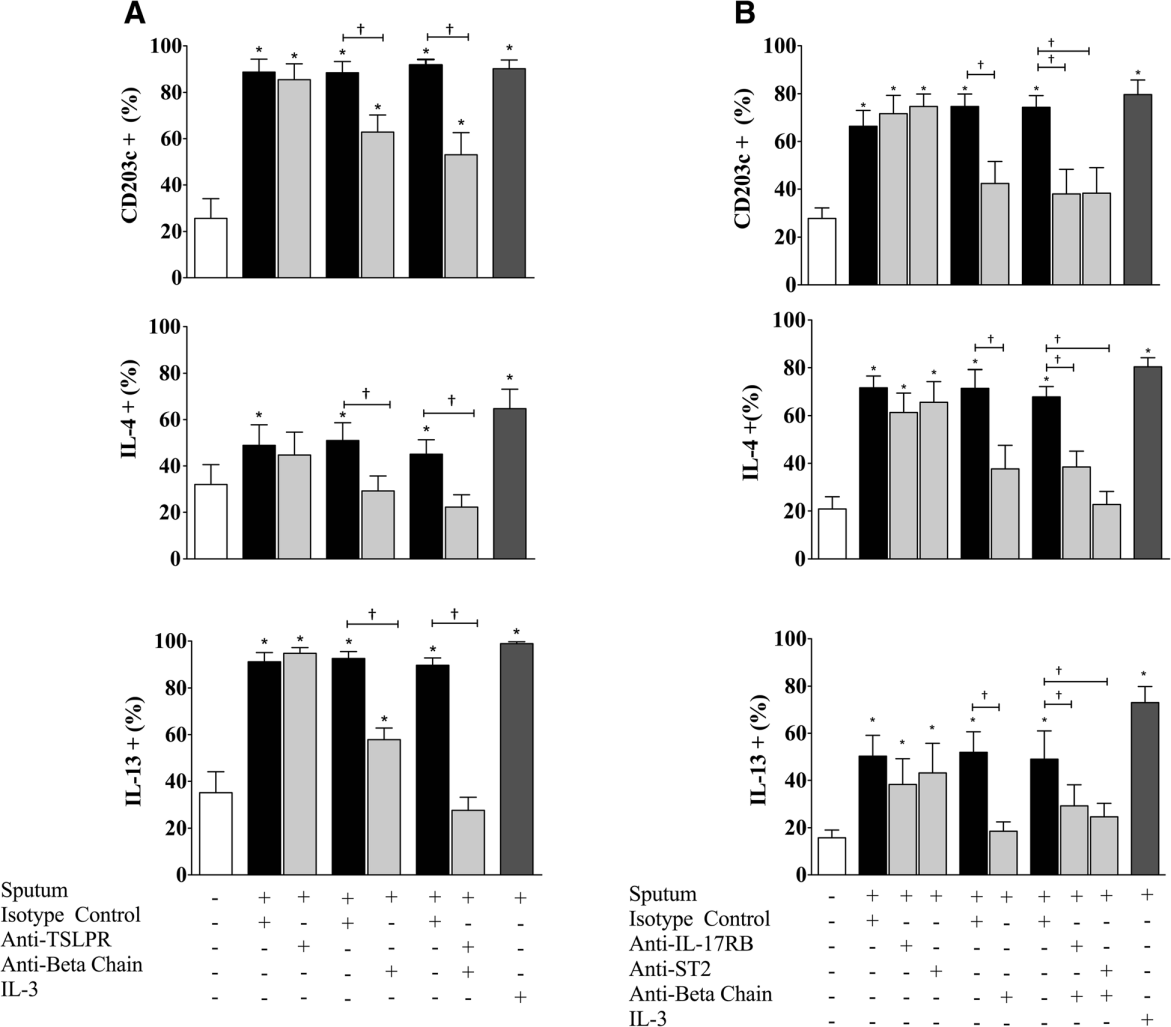
Blocking βc neutralizes the effects of airway samples on basophils. Purified peripheral blood basophils from 16 mild allergic asthmatic subjects were suspended in RPMI-C and cultured for 18 h with PBS as negative control (open bars) or 7 h post-allergen sputum supernatant and neutralizing antibodies to (**a**) TSLPR, (**b**) ST2 or IL-17RB, and (**a**, **b**) βc (10 μg/mL) (light grey bars) on markers of basophil function by expression of CD203c, IL-4, and IL-13. Data expressed as mean ± SEM. IL-3 (10 ng/mL) served as positive control (dark greys bars) *Significantly different from PBS negative control, † significantly different from isotype control (open bars); *P* < 0.05 (*N* = 8)

To ascertain the mechanisms through which the airway microenvironment promotes basophil activation post-allergen inhalation, we determined whether airway sample-induced basophil activation could be inhibited following treatment with neutralizing antibodies to the βc for IL-3/IL-5/GM-CSF, and the receptors for alarmin cytokines ST2, and IL-17RB. Blockade of the βc significantly inhibited basophil activation induced by 7 h post-allergen airway samples (Fig. 7). Treatment with neutralizing antibodies to TSLPR (Fig. 7a), ST2 (Fig. 7b), or IL-17RB (Fig. 7b) alone did not inhibit the stimulatory effects of 7 h post-allergen airway samples on markers of basophil activation. The addition of the anti-βc with anti-TSLPR, anti-ST2, or anti-IL-17RB significantly inhibited the stimulatory effects, but there was no synergistic effect (Fig. 7).

## Discussion

Previous studies have shown that basophils express ST2, and this can be up-regulated following in vitro stimulation with IL-33 or IL-3 [16, 31–34, 36]. We expanded on these findings by demonstrating that IL-25 and IL-33 up-regulated their own receptors on basophils. Interestingly, IL-3, anti-IgE, and TSLP stimulation up-regulated percent basophil expression of IL-17RB and ST2, however IL-25 and IL-33 had no effect on TSLPR, suggesting that these cytokines act downstream of TSLP. We have shown that the sensitivity of basophils to IL-33 and IL-25 can be up-regulated through both IgE-dependent or IgE-independent mechanisms.

Other studies have reported that IL-33 and IL-25 induce pro-inflammatory effects on human basophil activity [16, 31–34, 36]. IL-25 inhibits basophil apoptosis and promotes IgE-mediated degranulation, but does not induce IL-4 or IL-13 release [36], whereas IL-33 promotes basophil Type 2 cytokine production [16, 31–34]. Although Wang et al. did not find that IL-25 induced basophil Type 2 cytokine release, we observed an increase in percent basophil expression of intracellular IL-4 and IL-13. Discrepancies between these studies may be due to differences in time-points and methodologies. We confirm previous reports that overnight incubation of basophils with IL-33 increased the percent basophil expression of intracellular IL-4 and IL-13, but also showed up-regulation of IL-3Rα and CD203c expression. IL-25 and IL-33-induced basophil activity was inhibited following blockade of IL-17RB and ST2, demonstrating the effects of these cytokines are, in part, mediated directly through their own receptors.

Suzukawa et al. previously demonstrated that stimulation with IL-33 could enhance basophil migration towards eotaxin [33], however no studies have thus far have shown an effect of IL-25 on basophil migration. Using shape change as a surrogate marker for migration, as previously described [28, 29], we demonstrated that IL-25 and IL-33 prime basophil migratory potential to eotaxin through up-regulation of CCR3 expression. Conversely, Suzukawa et al. found no increase in basophil CCR3 expression following 1 h stimulation with IL-33, whereas we found that overnight IL-33 stimulation up-regulated CCR3 expression. Variation between these findings could be due to different stimulation time-points used. We have previously shown that following allergen inhalation, bone marrow basophils decrease in CCR3 expression at 24 h post-allergen, whereas CCR3 expression was up-regulated on both peripheral blood and sputum basophils [28]. Taken together with increased sputum basophil numbers post-allergen, this suggests that a subset of activated basophils expressing CCR3 influx to the lungs in response to eotaxin post-allergen challenge. This coincides with mechanisms of eosinophil migration from the bone marrow [40], with elevated peripheral eotaxin levels by 5 h post-allergen challenge [41] and with our findings that IL-25 and IL-33 up-regulate CCR3 basophil expression after overnight incubation.

Most importantly, we have shown up-regulation of percent basophil expression for IL-17RB within the bone marrow, peripheral blood and airways, as well as ST2 within the bone marrow and peripheral blood post-allergen challenge. These findings are similar to our previous reports that the percentage of basophils expressing TSLPR increases following allergen inhalation for up to 24 h [28]. Moreover, 24 h post-allergen percent basophil expression of ST2 positively correlated with allergen-induced bronchoconstriction. This is similar to our previous reports that peripheral blood percent basophil expression of TSLPR correlates with maximal % FEV_1_ fall during the LAR [28]. These findings suggest that interaction between basophils and alarmin cytokines, including TSLP, may be a contributing factor to declined lung function during the LAR. Basophils have been identified as a source of IL-25 following IgE cross-linking [35], and we found intracellular IL-25 expression to be elevated within the periphery and sputum post-allergen, which may further contribute to Type 2 inflammation. In contrast, we found no change in sputum percent basophil expression of ST2 following allergen challenge. We propose that this may be due to asthmatic subjects already having elevated ST2 expression pre-challenge due to the underlying disease, thereby making it difficult to detect changes in airway basophil expression. This is, in part, supported by Pecaric-Petkovic et al. who demonstrated that non-stimulated basophils in normal subjects had no detectable levels of ST2, but stimulation with IL-3 induced measurable levels [31]. Moreover, we have shown that a correlation between peripheral blood basophil ST2 expression and LAR bronchoconstriction.

The final component of this study was to examine basophil responsiveness to samples collected from the airway microenvironment during the LAR, containing mediators released by the epithelium and other inflammatory cells. Overnight incubation of isolated human basophils with 7 h post-allergen sputum supernatant yielded the greatest increase in percent basophil expression of CD203c, and intracellular IL-13 and IL-4. The 0 h airway samples also induced significant increases in percent cell expression of CD203c and intracellular IL-4, supporting the notion that allergic asthmatics have underlying Type 2 inflammation in the airways compared to healthy individuals. Collectively, these findings demonstrate that following allergen inhalation, the airway microenvironment can up-regulate activation markers on basophils. Blockade of βc led to inhibition of basophil activation induced by 7 h airway samples, whereas neutralization of receptors for alarmin cytokines had no inhibitory effect.

Murine models have shown that knock out of TSLPR, IL-17RB, or ST2 reduces AHR and eosinophilia, however blockade of these receptors in our experiments had no effect on basophil pro-inflammatory activity induced by airway samples. We have had difficulty detecting TSLP, IL-33 and IL-25 protein in sputum samples [28] and this may be due to alarmin cytokines being unstable and metabolized quickly, thus requiring more sensitive assays for measurement [42]. Previous studies have demonstrated that airway IL-33 and IL-25 mRNA expression is increased in asthmatics [23–26]. Furthermore, Fux et al. reported that IL-33 protein increases rapidly in BALF after allergen challenge, but declines to baseline levels at 18 h post-allergen [43]. It is possible that although alarmin cytokines are upstream triggers of Type 2 inflammation during the EAR, their levels may not be significant during the LAR. Downstream Type 2 cytokines such as IL-3, IL-5 and GM-CSF, may be more prevalent within the airways during the LAR. Therefore, blockade of βc has a more profound inhibitory effect on basophil activation in vitro, as opposed to epithelial-derived cytokine receptor neutralization. Lastly, basophils are known to have an IL-3 autocrine activation loop. We have previously reported that TSLP can induce basophil production of IL-3, which could in turn serve as another mechanism for TSLP to enhance basophil activation by signaling through IL-3Rα [28]. In this study we have demonstrated that IL-25 and IL-33-induced basophil activation was inhibited following blockade of IL-17RB and ST2, however, we cannot rule out the possibility that the effects of IL-25 and IL-33 may be mediated in an IL-3/IL-5/GM-CSF-dependent manner. Whether or not IL-25 and IL-33 exert their effects additionally through an IL-3 dependent mechanism will need to be elucidated further in the future.

We could not detect levels of IL-13 or IL-4 released from basophils due to the low numbers of cells utilized in these in vitro experiments. Instead, we measured intracellular cytokine expression through flow cytometry, however demonstrating intracytoplasmic expression of IL-13 and IL-4 cannot indicate whether these proteins are actually released [41]. Due to limited basophil numbers, migration experiments typically using trans-well migration were replaced with cell shape change as a surrogate measure of migration [44]. Although this is not the traditional method of determining migration, shape change is an effective way to assess migratory potential.

## Conclusion

In summary, we demonstrate that allergen inhalation increases airway basophil numbers and their sensitivity to IL-25 and IL-33 through up-regulation of the percentage of basophils expressing surface IL-17RB and ST2. Furthermore, markers of basophil activation significantly increase post-allergen in response to the airway microenvironment through an IL-3-dependent mechanism, which may be in part due to stimulation by IL-25 and IL-33. We conclude that basophil responses to these alarmin cytokines play an important role in potentiating Type 2 inflammation and serves as a novel therapeutic target for the treatment of allergic asthma.

## Additional files

Additional file 1: Figure S1. Bone marrow basophil gating strategy and change in marker expression. Representative bone marrow dot plots showing basophil gating strategy used for flow cytometry analysis including: (A) Side Scatter (SSC) vs. Forward Side Scatter (FSC), (B) SSC vs. CD45 Alexa Fluor 700, (C) SSC vs. HLA-DR APCH7 and (D) SSC vs. IL-3Rα PECy7. Panels E-G are representative dot plots of the change in the percentage of basophils expressing IL-17RB, ST2, and intracellular IL-25 at pre-allergen challenge (blue) and 24 h post-allergen challenge (green), compared to the isotype control (red). (TIFF 10587 kb) Additional file 2: Figure S2. Peripheral blood basophil gating strategy and change in marker expression. Representative peripheral blood dot plots showing basophil gating strategy used for flow cytometry analysis including: (A) SSC vs. FSC, (B) SSC vs. CD45 Alexa Fluor 700, (C) SSC vs. HLA-DR APCH7 and (D) SSC vs. IL-3Rα PECy7. Panels E-G are representative dot plots of the change in the percentage of basophils expressing IL-17RB, ST2, and intracellular IL-25 at pre-allergen challenge (blue), 7 h post-allergen (green) and 24 h post-allergen challenge (orange), compared to the isotype control (red). (TIFF 9034 kb) Additional file 3: Figure S3. Sputum basophil gating strategy and change in marker expression. Representative sputum dot plots showing basophil gating strategy used for flow cytometry analysis including: (A) Side Scatter (SSC) vs. Forward Side Scatter (FSC), (B) SSC vs. CD45 Alexa Fluor 700, (C) SSC vs. HLA-DR APCH7 and (D) SSC vs. IL-3Rα PECy7. Panels E-G are representative dot plots of the change in the percentage of basophils expressing IL-17RB, ST2, and intracellular IL-25 at pre-allergen challenge (blue), 7 h post-allergen (green) and 24 h post-allergen challenge (orange), compared to the isotype control (red). (TIFF 10198 kb) Additional file 4: Figure S4. Purified basophil gating strategy for in vitro cultures. Representative dot plots demonstrating gating strategy used for flow cytometry analysis of purified basophils including: (A) SSC vs. CD45 Alexa Fluor 700 (B) SSC vs. HLA-DR APCH7 and (C) SSC vs. IL-3Rα PECy7. Purified basophil populations had a mean purity of >98 %. (TIFF 3300 kb) Additional file 5: Figure S5. Basophil activation marker dose response to IL-33. Isolated peripheral blood basophils from 8 allergic subjects were suspended in RPMI-C and cultured for 18 h with PBS (negative control, open bars) or increasing concentrations of IL-33 (0.1, 1, 10, 100 ng/mL) (black bars), were assessed for expression of (A) CD203c, (B) IL-3Rα, (C) CCR3, (D) intracellular IL-13, (E) intracellular IL-4, (F) intracellular IL-25, (G) IL-17RB, (H) ST2, and (I) TSLPR. Data expressed as mean ± SEM. *Significantly different from PBS negative control; *P* < 0.05 (*N* = 8). (TIFF 2756 kb) Additional file 6: Figure S6. Basophil activation marker dose response to IL-25. Isolated peripheral blood basophils from 8 allergic subjects were suspended in RPMI-C and cultured for 18 h with PBS (negative control, open bars) or increasing concentrations of IL-25 (0.1, 1, 10, 100 ng/mL) (black bars), were assessed for expression of (A) CD203c, (B) IL-3Rα, (C) CCR3, (D) intracellular IL-13, (E) intracellular IL-4, (F) intracellular IL-25, (G) IL-17RB, (H) ST2, and (I) TSLPR. Data expressed as mean ± SEM. *Significantly different from PBS negative control; P < 0.05 (N = 8). (TIFF 2740 kb)

## Abbreviations

βc: Common β-chain
CCR3: Chemokine Receptor-3
EAR: Early Asthmatic Response
FACS: Fluorescence-activated cell sorting
FSC: Front angle light scatter
H: Hour
IL: Interleukin
IL-17RB: IL-25 Receptor
LAR: Late Asthmatic Response
mAb: monoclonal antibody
MCP: monocyte chemoattractant protein
MIP: macrophage inflammatory protein
Min: Minute
S: Second
SSC: Side angle light scatter
ST2: IL-33 Receptor
RT: Room Temperature
TSLP: Thymic stromal lymphopoietin
