# Supplementary figures and images for: IL-25 and IL-33 induce Type 2 inflammation in basophils from subjects with allergic asthma
Source: Respir Res. 2016 Jan 14;17:5. doi: 10.1186/s12931-016-0321-z (PMC4712475; doi:10.1186/s12931-016-0321-z)

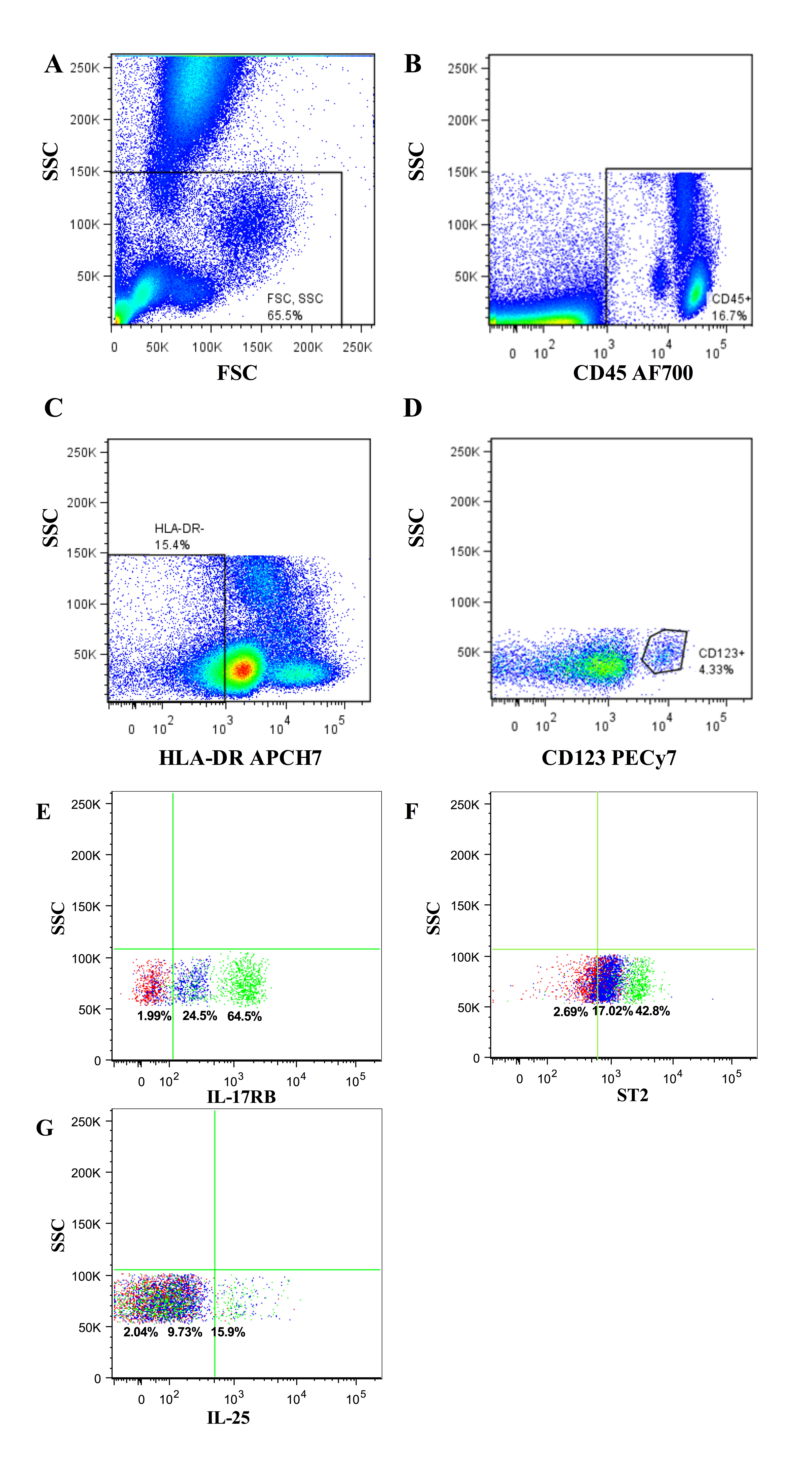

Supplement: Additional file 1: Figure S1. — Bone marrow basophil gating strategy and change in marker expression. Representative bone marrow dot plots showing basophil gating strategy used for flow cytometry analysis including: (A) Side Scatter (SSC) vs. Forward Side Scatter (FSC), (B) SSC vs. CD45 Alexa Fluor 700, (C) SSC vs. HLA-DR APCH7 and (D) SSC vs. IL-3Rα PECy7. Panels E-G are representative dot plots of the change in the percentage of basophils expressing IL-17RB, ST2, and intracellular IL-25 at pre-allergen challenge (blue) and 24 h post-allergen challenge (green), compared to the isotype control (red). (TIFF 10587 kb) [file 12931_2016_321_MOESM1_ESM.tiff]

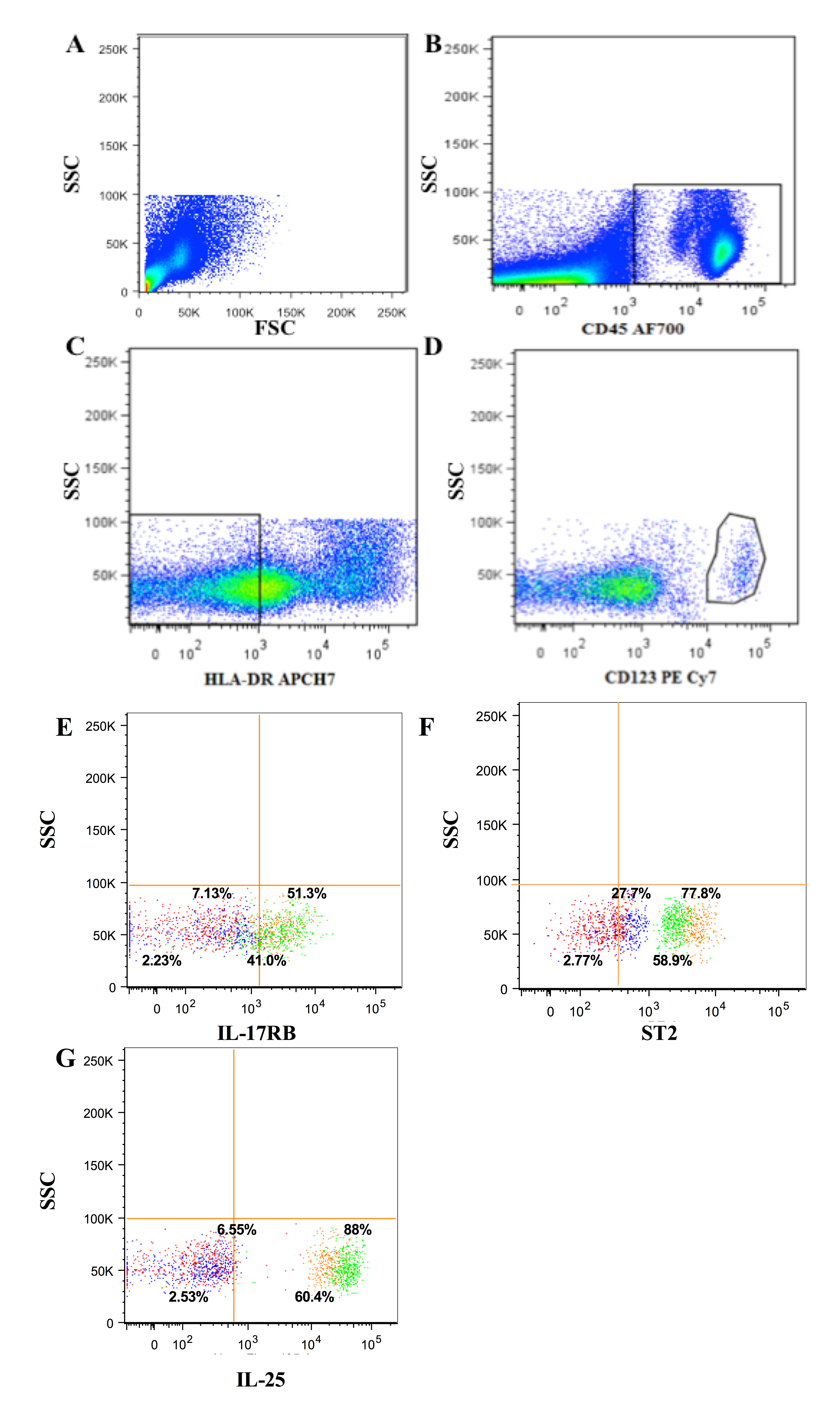

Supplement: Additional file 2: Figure S2. — Peripheral blood basophil gating strategy and change in marker expression. Representative peripheral blood dot plots showing basophil gating strategy used for flow cytometry analysis including: (A) SSC vs. FSC, (B) SSC vs. CD45 Alexa Fluor 700, (C) SSC vs. HLA-DR APCH7 and (D) SSC vs. IL-3Rα PECy7. Panels E-G are representative dot plots of the change in the percentage of basophils expressing IL-17RB, ST2, and intracellular IL-25 at pre-allergen challenge (blue), 7 h post-allergen (green) and 24 h post-allergen challenge (orange), compared to the isotype control (red). (TIFF 9034 kb) [file 12931_2016_321_MOESM2_ESM.tiff]

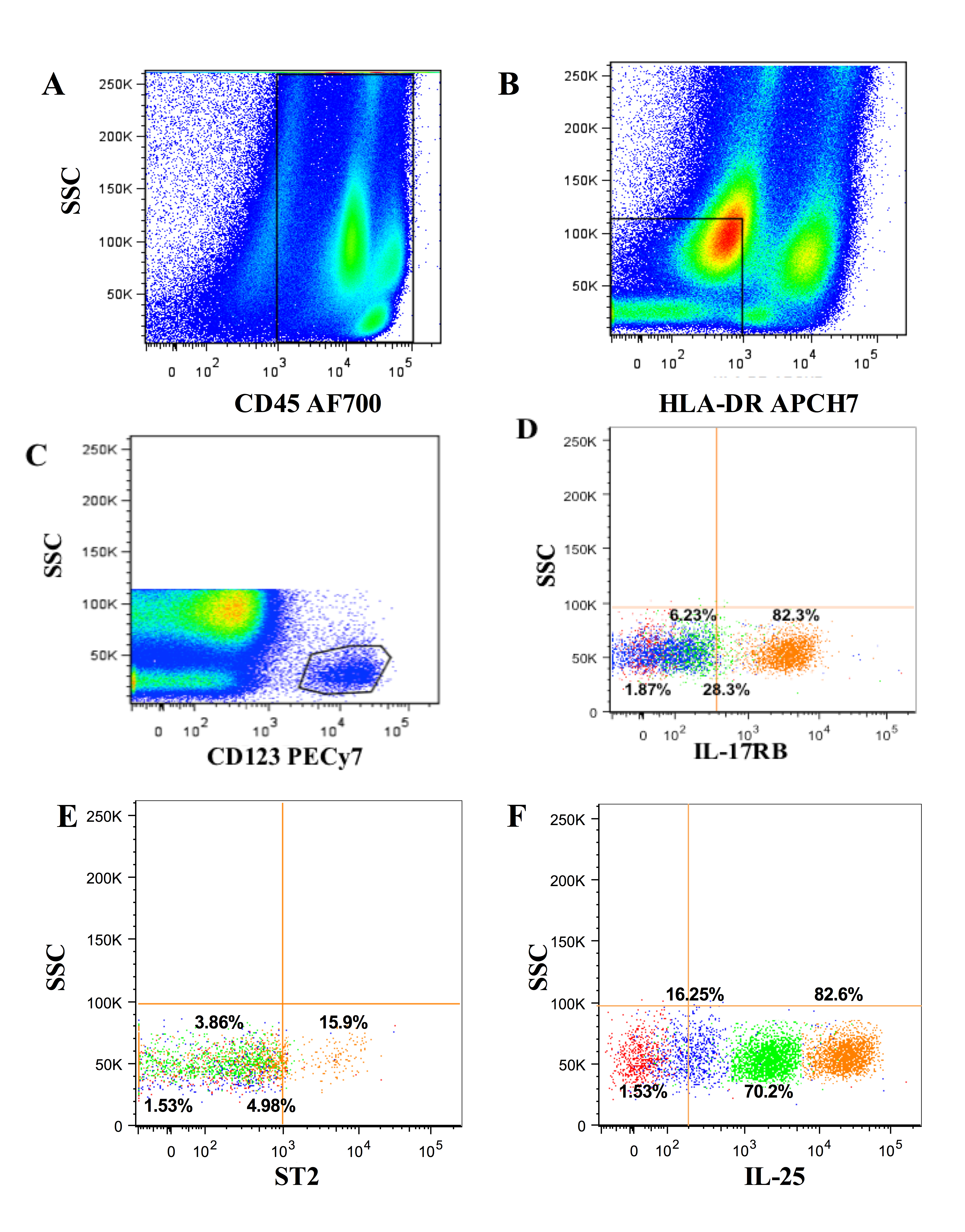

Supplement: Additional file 3: Figure S3. — Sputum basophil gating strategy and change in marker expression. Representative sputum dot plots showing basophil gating strategy used for flow cytometry analysis including: (A) Side Scatter (SSC) vs. Forward Side Scatter (FSC), (B) SSC vs. CD45 Alexa Fluor 700, (C) SSC vs. HLA-DR APCH7 and (D) SSC vs. IL-3Rα PECy7. Panels E-G are representative dot plots of the change in the percentage of basophils expressing IL-17RB, ST2, and intracellular IL-25 at pre-allergen challenge (blue), 7 h post-allergen (green) and 24 h post-allergen challenge (orange), compared to the isotype control (red). (TIFF 10198 kb) [file 12931_2016_321_MOESM3_ESM.tiff]

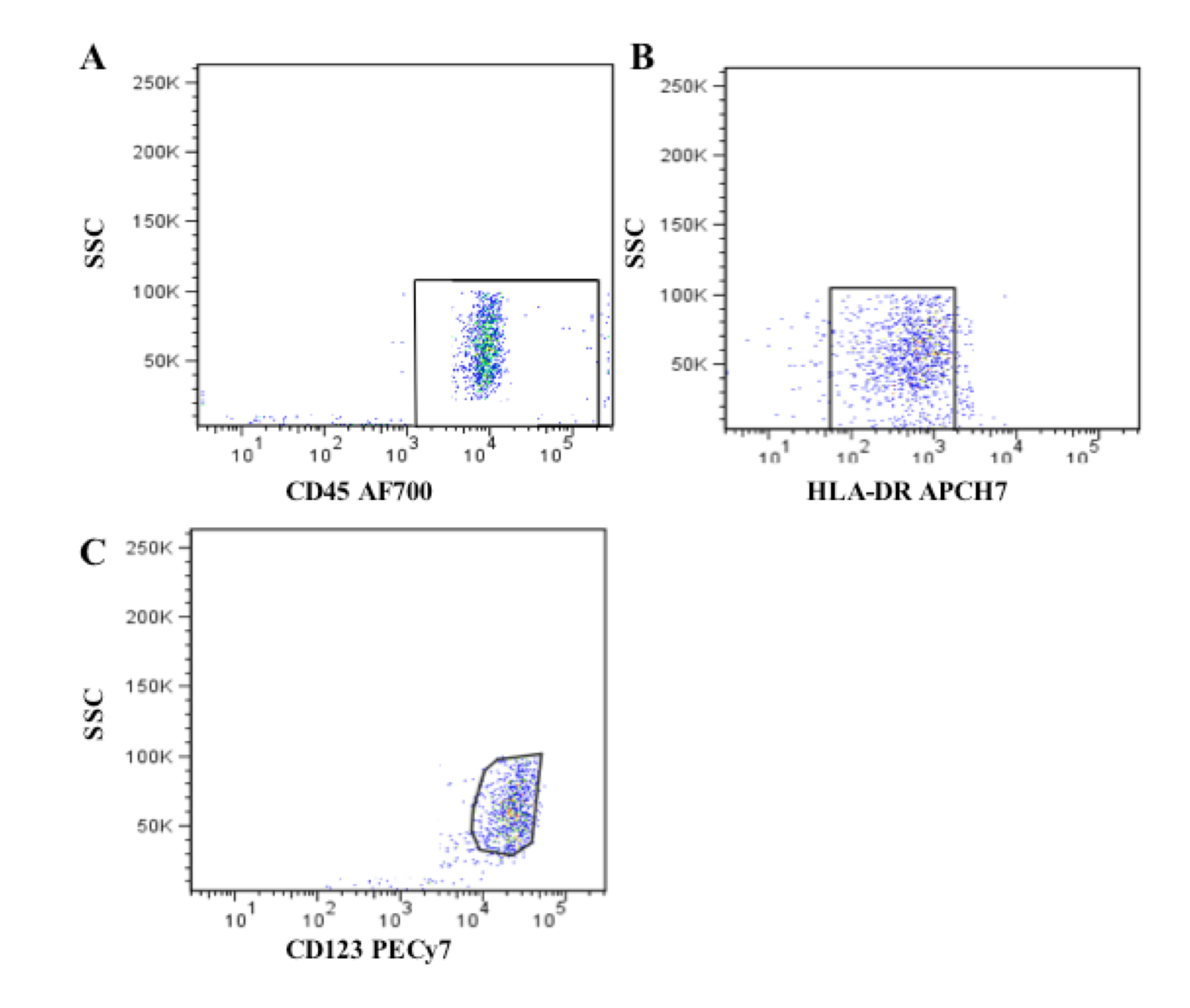

Supplement: Additional file 4: Figure S4. — Purified basophil gating strategy for in vitro cultures. Representative dot plots demonstrating gating strategy used for flow cytometry analysis of purified basophils including: (A) SSC vs. CD45 Alexa Fluor 700 (B) SSC vs. HLA-DR APCH7 and (C) SSC vs. IL-3Rα PECy7. Purified basophil populations had a mean purity of >98 %. (TIFF 3300 kb) [file 12931_2016_321_MOESM4_ESM.tiff]
